# Using Latent Class Analysis to Identify Need Typologies and Recidivism Likelihood Among Women Who Perpetrate Violence

**DOI:** 10.1177/08862605241246008

**Published:** 2024-04-29

**Authors:** Lauren Belyea, Shelley L. Brown, Marilyn Van Dieten

**Affiliations:** 1 Carleton University, Ottawa, ON, Canada; 2 Center for Effective Public Policy, Silver Spring, MD, USA

**Keywords:** domestic violence, intimate partner violence, impacted women, violence, violent women

## Abstract

Understanding the heterogeneity of women who engage in violence is critical to provide effective treatment and reduce the likelihood of recidivism. Existing typologies of women who engage in violence have been created using mixed methodological approaches; the field would benefit from replication using a quantitative clustering method—latent class analysis (LCA)—as it is arguably more objective than methods used to date. A LCA was conducted using archival data involving 3,773 justice-impacted women in Western Canada to identify unique subgroups of women who perpetrate violence. Three distinct profiles emerged: (a) intimate partner violence (IPV)-only (40.5%), wherein almost all women reported only perpetrating domestic violence and had zero or only one previous violent conviction, (b) patterned (19.1%), wherein violence was perpetrated toward domestic partners and unknown victims, and the majority had two or more previous violent convictions, and (c) isolated (40.4%), wherein very few perpetrated domestic violence, some perpetrated violence toward unknowns, and the majority had either zero or only one previous conviction for a violent offense. Need profiles and recidivism outcomes were further analyzed as a function of group membership. As hypothesized, the group with the greatest criminal history and use of violence reported the greatest needs. Recidivism also increased as the number of dynamic needs increased. Notably, 80.9% of the sample was predominantly low risk/low need and were identified as IPV-only or isolated women. Implications of these findings may be used to inform risk classification, treatment targets, and treatment intensity required to reduce the likelihood of recidivism among women who perpetrate violence.

Since 1980, the number of women under correctional control in the United States has increased twofold relative to men (Monazzam & Budd, 2023). Even the most recent data illustrates that while incarceration rates are again climbing since the COVID-19 decline of 2020, the incarceration of women (3.6% increase from 2021 to 2022) is 2.5 times higher relative to men (1.5% increase from 2021 to 2022) (Carson, 2023). Consequently, for the last four decades, feminist-inspired researchers have been examining the reasons for mass incarceration, how to reduce it, how to best meet the needs of women while incarcerated, and lastly, how to best reduce barriers to re-entry (Adler, 1975; Belknap, 1995, 2021; Blanchette & Brown, 2006; Bloom et al., 2003; Chesney-Lind, 1989; Daly, 1992; Van Voorhis, 2012, 2022).

The expansive body of feminist-inspired scholarship has at times viewed justice-impacted women as a homogenous group, failing to consider potential differences in needs and other contributing factors that underpin female-perpetrated violence, in particular. Learning more about the different risk/need profiles of women who perpetrate violence and how these profiles relate to recidivism outcomes is necessary to inform theory, risk assessment, and treatment. Further, the sparse research to date identifying typologies of women who engage in violence has used different (and at times unclear) methodological approaches and different sampling frames for typology development (see Van Dieten et al., 2014 for a review). It remains to be verified if such typologies would also emerge if derived using newer quantitative clustering techniques such as latent class analysis (LCA); arguably, LCA is less subjective than some methods used to date. LCA is much like factor analysis. Factor analysis identifies a reduced set of unobservable (latent) over-arching thematically related variables (known as factors) from a larger set of observed indicators using statistical methods. LCA performs the same task. However, instead of extracting a set of latent variables, LCA extracts clusters of “latent” people characterized by similar traits and/or behaviors. As such, the goal of the study is to (a) explore if a heterogenous latent violence typology of women exists, (b) if latent class membership is best characterized by a matter of degree—more or less violence, or by a matter of kind—emergence of different profiles of women with unique patterns of violence (e.g., intimate partner violence [IPV] only vs. patterned violence in multiple situations), and (c) if the resultant latent classes can be further differentiated in terms of adverse childhood experiences (ACEs), risk level, need profiles, and recidivism likelihood.

Of the over 1.7 million offenses committed by women in the United States in the year 2020, the most frequent were assault (31.1%), followed by larceny/theft (22.5%), and drug/narcotic offenses (21.0%) (Uniform Crime Reporting Program, 2020). Similarly, in Canada, the most recent report at the time of writing demonstrated that 27% of all female-accused crimes were violent in nature (Department of Justice Canada, 2020). This figure has remained consistent in Canada across previous years. In 2017, 25% of female-accused crimes were classified as violent (Savage, 2019); the vast majority (70%) of these violent crimes were assault-related. Of these assault crimes, over three-quarters (76%) were classified as the least serious, simple assault (Savage, 2019), indicating that little to no harm was caused to the victim. Other female-accused violent crimes were uttering threats (11.1%), criminal harassment (4.9%), and robbery (3.1%). Consistent with the theoretical nature of women’s offending, wherein violence is predominantly relational (Brzozowski et al., 2018), most women accused of violent crimes in Canada knew their victim (Savage, 2019). In cases concerning a single victim and perpetrator, women most often perpetrated violence against an intimate partner (36%) or a casual acquaintance (22%; Savage, 2019). Taken together, evidence suggests that when women perpetrate violence, it is most likely assault toward someone they know.

Several factors have been identified which may initiate and maintain women’s justice system involvement, including women who have engaged in violence. Many of these factors emerged within feminist pathways theory—arguably, the genesis of trauma-centered scholarship in the context of impacted women (and girls). Feminist pathways theory arose in the 1980s with the seminal works of authors such as Miller (1986), Chesney-Lind (1989), and Daly (1992). These early theorists were among the first to explicitly document the impact of childhood abuse and childhood adversities as a pathway to the justice system (e.g., using substances to cope with the impact of abuse).

The 1980s and 1990s bore witness to numerous retrospective qualitative studies that continued to support feminist pathways theory (see Belknap, 2021; Holtfreter et al., 2022 for reviews). However, the pathways studies of the 1980s and 1990s provided more nuanced insights illuminating how abuse and trauma can translate into criminal justice involvement. Notably, Daly’s (1992) now classic life history analysis of pre-sentence reports for 40 women identified 5 paths to the justice system: *street*, *harmed and harming*, *battered*, *drug connected*, and *economically motivated.* While the street and harmed/harming women fit the “leading feminist scenario of lawbreaking” (Daly, 1992, pp. 13–14)—that being, childhood abuse as the causal factor—Daly’s analysis also illustrated how the sequalae of childhood abuse (e.g., using substances to cope) and harmful relationships with intimate partners (or family members) can also contribute to women’s justice involvement. Notably, Daly identified a group of battered women whose first contact with the justice system was the direct result of being in a relationship with a violent man.

The pioneering work of feminist pathways theorists has been validated and expanded using quantitative typology methods (e.g., Brennan et al., 2012; Brennan & Jackson, 2022; Brown et al., 2021; DeHart, 2018; Reisig et al., 2006; Salisbury & Van Voorhis, 2009), and developmental life course trajectory modeling (e.g., Broidy et al., 2018; Piquero et al., 2022). Collectively these analyses have re-affirmed and expanded upon the original pathway’s findings. Specifically, research has re-affirmed the role that childhood abuse, childhood adversity, and parental supervision practices play in catapulting girls and women (and men and boys) toward the justice system. These analyses have also underscored that it is often the sequalae of abuse and adversities, namely, complex trauma symptomology—post-traumatic stress disorder (PTSD), substance abuse, poor affect regulation, relational mistrust (and unhealthy attachment styles), mental health challenges, and economical marginalization that contribute more proximally to women and girls becoming justice impacted (Brown et al., 2021; Gehring, 2018). Notably, quantitative typology researchers have also identified a battered woman profile (Brennan et al., 2012; Burgess-Proctor et al., 2016; Simpson et al., 2008; Smith, 2017).

Typology-building research specific to women who have perpetrated violence remains sparse, however. Nonetheless, seminal work in this area does exist and has laid the ground work for our study. First, Babcock et al.’s (2003) conducted an influential study exploring potential differences among women arrested for domestic violence. The authors studied 60 women who opted to attend an intervention agency after their domestic violence arrest. The authors classified the women as either generally violent (*n* = 30) or partner-only (*n* = 30). Women categorized as generally violent had reported using IPV in the year prior and violence against a non-intimate since age 18 (e.g., family members, friends, and police officers), whereas partner-only women had not used violence toward a non-intimate since age 18 and had directed violence exclusively toward an intimate partner in the year prior. The average age of the sample was 31.5, with over half (54%) indicating they were White (24% Latina, 17% Black).

Overall, women categorized as generally violent reported using greater frequency and severity of violent acts [i.e., physical abuse, abuse which caused partner injury; Conflict Tactics Scale 2 [CTS2]; Straus et al. (1996)], as well as emotionally abusive tactics (i.e., psychological abuse; CTS2), than partner-only women, despite both groups reporting a similar number of domestic and non-domestic previous arrests. Women categorized as generally violent also endorsed using violence to attain a goal (i.e., instrumental), such as controlling their partner and externalized blame for their violence more often (i.e., blame partner or a lack of self-control). Moreover, despite similar reports of childhood physical and sexual victimization between groups, women categorized as generally violent reported greater overall traumatic symptoms (i.e., greater desire to hurt themselves or others, as well as interpersonal and memory problems), as well as witnessing their mothers aggress against their fathers in childhood more often.

Years later, Kubiak et al. (2013) examined within-group differences in a sample of 417 women incarcerated in a Midwestern state prison. Using official conviction history and self-reported violence in the prior 12 months, the women were categorized using the following researcher-defined decision rules: (a) uncaught women who engaged in violence but were serving time for a nonviolent offense (*n* = 52), (b) isolated women who were convicted of a single violent offense but hadn’t engaged in any additional violence (*n* = 172), and (c) patterned women who were both convicted of a violent offense and engaged in uncaught violence (*n* = 193). Participant demographics and offense information were not collected.

Overall, the uncaught and patterned groups reported many similarities, whereas the isolated groups differed greatly. In terms of criminal history, uncaught and patterned women were significantly more likely than isolated women to have been previously arrested, convicted, or incarcerated in a state facility, though, notably, a large proportion of isolated women were serving sentences for homicide. Although no group differences were observed in terms of IPV victimization history (i.e., severe physical acts by partner, intimidation by partner), uncaught and patterned women were more likely to report a substance dependence, previous mental health treatment, and use of psychiatric medication than isolated women. Uncaught and patterned women also reported significantly greater rates of instrumental and expressive anger, impulsivity, and inhibition (i.e., getting into conflict while misusing alcohol/drugs), as well as higher overall risk, than isolated women. Kubiak et al. did not measure whether the women had engaged in IPV themselves, however.

Kubiak et al.’s (2013) findings show some similarities with Babcock et al. (2003). Despite using different measures, both studies reported similar victimization histories (IPV or childhood) within the samples. As well, both authors identified a group of women who had engaged in an isolated incident of violence and were characterized by less psychopathology (e.g., less desire to hurt themselves/others, less mental health needs). Additionally, both authors identified a second group of women characterized by multiple incidents of violence and more psychopathology and treatment needs relative to the women characterized as isolated. As both studies used researcher-derived decision rules to manually categorize women based on their use of violence, it remains unknown if these group characteristics would also emerge using a formal quantitative clustering technique.

Based on individual risk and need assessments, Fatania (2010) used Smallest Space Analysis, a multidimensional scaling technique for quantitative clustering, with a sample of women (77.7% White, 18.6% missing data) incarcerated for an IPV-related offense in the United Kingdom. The analysis revealed two distinct groups of women: high-moderate criminality and high-moderate psychopathology (*n* = 30), and high-moderate psychopathology and low-moderate criminality (*n* = 170). In terms of criminal history, women in Fatania’s (2010) high-moderate criminality and high-moderate psychopathology group reported significantly more previous custodial sentences and breaches relative to the low-moderate criminality group; this is similar to Kubiak et al.’s (2013) patterned and uncaught groups. The high-moderate criminality and high-moderate psychopathology group also demonstrated greater exposure to extra-familial violence, young behavioral problems, pro-criminal attitudes, recklessness, inadequate interpersonal skills, aggression, anger, impulsivity, and substance abuse than the other group. Such heightened psychopathology is consistent with Babcock et al.’s (2003) generally violent group and Kubiak et al.’s uncaught and patterned groups.

In contrast, Fatania (2010)’s high-moderate psychopathology and low-moderate criminality group reported greater history of alcohol misuse, depression, suicide/self-harm, weapon use, and current psychiatric problems and treatment. This group differed from typologies put forward by Babcock et al. and Kubiak et al., as heightened psychopathology was only identified in Babcock et al.’s group with heightened use of violence and Kubiak et al.’s groups with heightened criminal history. Such differences among studies may be attributed to Fatania’s use of multidimensional scaling methodology; similar groupings may not have emerged in the noted studies for methodological reasons, including the use of different measures, samples (e.g., some only comprised of IPV women only), as well as the common practice of using researcher-derived a priori decision rules to manually assign women to specific typological groups.

Altogether, more research is needed to examine if the typologies of women who perpetrate violence found in past studies will also emerge using an increasingly popular quantitative clustering technique (latent cluster analysis) with demonstrated high statistical rigor (Nylund-Gibson & Young Choi, 2018). Typologies of women categorized as violent also need to be assessed more broadly rather than restricted to women who perpetrate IPV (i.e., Babcock et al., 2003; Fatania, 2010). This is particularly important in practice as women charged with violent crimes are more likely to be classified as higher risk without regard to the context of the crime and the historical use of violence. In turn, this can impact how women are classified within facilities, which elevates the likelihood of placement in restrictive housing. Similarly, high-risk women serving sentences in the community are more likely to receive more intensive supervision and surveillance from probation and parole officers. Ultimately, women charged with violent crimes are less likely to participate in treatment and vocational programming and may not be considered eligible for diversion programs. Noteworthy, diversion programs are typically used in Canada and the United States to resolve minor crimes and avoid conviction for the offense. Diversion programs often require the individual to complete a treatment program(s), perform community services, or make donations to avoid official criminal persecution in a court of law.

In line with the risk-need-responsivity (RNR) model (Bonta & Andrews, 2024), and gender-responsive approaches (Belknap, 2021; Bloom et al., 2003; Covington, n.d.), risk/need information can be used to: (a) target factors which contribute to, and maintain offending behavior (e.g., ACEs, dynamic needs), (b) determine optimal treatment intensity (e.g., admission to a time-intensive clinical inpatient program for higher risk cases vs. a less time-intensive outpatient intervention for lower risk cases), and (c) adopt the best person-specific approach (e.g., learning style, motivation, ability, trauma-informed). Such approaches are designed to maximize recidivism reductions. Inappropriate matching of treatment targets/intensity with an impacted woman’s needs and failure to address contributing trauma symptomology (e.g., resulting from ACEs) not only misuses resources but may also reduce the effectiveness of such treatment approaches (Fritzon et al., 2021) and inadvertently increase criminal behavior (Andrews & Bonta, 2006, 2010).

There is evidence that cognitive behavioral treatments blended with gender-responsive principles can reduce recidivism in mainstream samples of impacted women, particularly programs designed to target substance misuse (Gobeil et al., 2016). However, few empirically validated intervention programs have been developed specifically for women who commit IPV or engage in other forms of violent behavior [for an exception, see Kubiak et al. (2016) for an evaluation targeting women who have committed violent crimes]. Also noteworthy, women charged with IPV are frequently mandated to batterer programs designed for men (Dutton et al., 2005). This continues to be the dominant practice, despite the absence of positive outcome evaluations with men (Babcock et al., 2004; Wilson et al., 2021) or the complete absence of women-specific evaluations. There is also evidence that mandated batterer programs may be contraindicated for women (Buttell, 2002). This is not surprising given that most mandated battered programs are either fully or partially grounded in the Duluth Model—a feminist-grounded approach designed specifically for men who perpetrate IPV; the Duluth Model attempts to change patriarchal cognitions toward women and unlearn power and control motivations (Pence et al., 2011). Thus, research that serves to better understand the heterogeneity of women who perpetrator violence, including women who perpetrate IPV has important treatment implications.

## Study Objectives and Hypotheses

Though the noted studies provide groundwork for different typologies of women who perpetrate violence, expansion and replication in the current landscape are critical to build insight into the antecedents, frequency, and severity of women’s use of violence, both IPV and non-IPV. Additional investigation of women’s risk/need profiles, ACEs, and recidivism likelihood as a function of group membership is also necessary to inform effective intervention for different types of women who perpetrate violence. The study examined the heterogeneity of justice-impacted women categorized as violent as a function of their criminal history, use of aggression/violence, risk/need profiles, and recidivism outcomes. Although theoretical and empirical support exists for differing typologies of women categorized as violent, such research has predominantly used researcher-defined decision rules grounded in theory (i.e., Babcock et al., 2003; Kubiak et al., 2013) or has been restricted to women convicted of an IPV-related offense (i.e., Fatania, 2010). The main goal was, therefore, to expand past research by using a comprehensive risk/need assessment of a large-scale population of women convicted for violent offenses in Western Canada to identify latent subgroups; the resultant, statistically-derived categories may be used to inform treatment planning and approaches to manage risk. Data collected using the Service Planning Inventory (SPIn; Orbis Partners, 2003), a risk-assessment tool for justice-impacted adults, was used in the LCA.

Based on past literature, we hypothesized that three typologies would emerge based on women’s use of aggression/violence: IPV-only (i.e., violence toward intimates only), patterned (i.e., violence toward intimates and non-intimates), and isolated (i.e., no violence toward intimates, but some other use of violence). As most women’s use of violence is relational in nature, we hypothesized that a large proportion of the sample would be classified as IPV-only. The second goal was to examine how individual need profiles and ACE scores mapped onto the resultant classes, and recidivism outcomes. Based on past studies, we hypothesized that the group with the greatest criminal history and/or use of violence, patterned women, would report the greatest dynamic needs and highest ACE scores. In line with RNR principles, we further expected the group with the greatest dynamic needs to report the greatest likelihood of recidivism.

## Methods

### Participants

The study used a de-identified archival SPIn (Orbis Partners, 2003) dataset (*N* = 31,477) provided by Orbis Partners Inc., a Canadian consulting firm that designs and implements services (e.g., evidence-based interventions) for clients at risk for justice involvement. The dataset was filtered using the following inclusion criteria: (a) women only and (b) the presence of at least one conviction or adjudication for a past or current violent offense from the SPIn criminal history domain, and/or at least one static indicator of violent behavior from the SPIn aggression/violence domain (see Tables 1 and 2).

**Table 1.** Descriptive Statistics Derived from SPIn.

Indicators Used in Latent Class Analysis^a^	*n*	%
Assault/Violent offense conviction	2,698	71.5
Violent behavior in last 6 months	1,856	49.2
Previous convictions for violent offense		
1	1,813	48.1
2	431	11.4
3+	400	10.6
Any violence toward unknown victims	746	19.8
Perpetrator of domestic violence	1,852	49.1
Violations of protection or no contact orders^b^	318	8.4
Any violent behavior before 16^c^	431	11.4
Weapons offenses^d^		
1	415	11.0
2	64	1.7
3+	16	0.4
Any history of violence associated with mental disordere	229	6.1

*Note*. SPIn = Service Planning Inventory.

a All indicators based on official conviction records or other credible reports.

b 0.0% missing data (*n* = 1).

c 23.6% missing data (*n* = 890).

d 27.7% missing data (*n* = 1,044).

e 29.2% missing data (*n* = 1,100).

**Table 2.** SPIn Descriptive Statistics.

SPIn Dynamic Need Domain	Overall Rating							
	None		Low		Moderate		High	
	*n*	%	*n*	%	*n*	%	*n*	%
Substance use	1,250	33.1	703	18.6	1,557	41.3	263	7.0
Social influences	1,384	36.7	751	19.9	1,078	28.6	560	14.8
Family	561	14.9	803	21.3	1,412	37.4	997	26.4
Employment	2,400	63.6	636	16.9	500	13.3	237	6.3
Attitudes	2,309	61.2	958	25.4	355	9.4	151	4.0
Social/Cognitive skills	2,731	72.4	594	15.7	239	6.3	209	5.5
Stability	1,556	41.2	1,004	26.6	917	24.3	296	7.8
Mental health (static and dynamic flags)	N/A	N/A	1,446	38.3	644	17.1	1,686	44.6
SPIn pre-screen overall risk classification^a^	N/A	N/A	1,999	53.0	1,460	38.7	314	8.3

Note. SPIn = Service Planning Instrument.

a Includes static and dynamic risk.

A final sample of 3,773 justice-involved women who began community supervision in Western Canada between 2009 and 2012 were used in our study. All women were serving a provincial sentence for an index offense (s); participants either began community supervision immediately (e.g., on probation or a community-based conditional sentence) or started community supervision post-release (i.e., on parole following a carceral sentence less than 2 years). At the time of the original SPIn assessment, the age range of participants was 10 to 80 (*M* = 31.71, *SD* = 10.15; Mdn = 30).^1^ A 3-year follow-up period from time of original assessment was used to evaluate recidivism.

In terms of racial background, participants were classified as non-Indigenous (58.4%, *n* = 2,203), Indigenous (37.2%, *n* = 1,404), or Other (4.4%, *n* = 166). SPIn pre-screen risk assessment (see Measures section), data indicated that half of the samples were classified as low risk (53%, *n* = 1,999), 38.7% were moderate risk (*n* = 1,460), and 8.3% were classified as high risk (*n* = 314). At original assessment, most participants had been convicted of a violent offense (71.5%, *n* = 2,698) and had at least one previous conviction for a violent offense (70.1%, *n* = 2,644). SPIn assessments revealed that approximately half of the sample had engaged in some form of violent behavior (that they may not have necessarily been charged or convicted for) in the last 6 months (49.2%, *n* = 1,856), and/or they had been convicted of IPV or admitted to engaging in IPV (49.1%, *n* = 1,852); few participants had engaged in violence toward unknown victim(s) (19.8%, *n* = 746).

### Measures

#### Service Planning Instrument (SPIn)

The SPIn (Orbis Partners, 2003) is an assessment and case management tool used to assess the risk/need/strength profiles of justice-impacted adults. Various risks, needs, and strengths are assessed using official, collateral, and self-report information to inform risk classification as well as individualized service and treatment plans for those in custody and community settings. The SPIn assesses both static and unchangeable factors, as well as dynamic factors that are changeable and can be targeted with intervention (Jones & Robinson, 2017).

The SPIn has demonstrated inter-rater reliability and predictive validity (Brown et al., 2020b). The SPIn full assessment consists of 90 static and dynamic items across 11 domains; 35 of these items are also used to calculate a Pre-Screen risk score. The full assessment domains are (a) criminal history; (b) response to supervision, such as technical violations or breaches; (c) aggression/violence, including domestic violence and weapon offenses; (d) substance abuse, which is any alcohol/drug use that causes life functioning disruption; (e) social influences, including gang associations, antisocial peers, and neighborhood; (f) family, such as marital risk factors and parenting skills; (g) employment, including employment history, motivation, and marketability; (h) attitudes, such as law-abiding attitudes, readiness for change; (i) social/cognitive skills, including impulsivity, and positive interpersonal skills; (j) stability, such as financial, accommodations, and life skills; and (k) mental health, including flags for mental health conditions, suicidal ideation, and trauma/abuse. Items within each domain are classified as either static risk, static protective, dynamic need, or dynamic protective.

Aggregate scores within each SPIn domain are calculated by separately summing weighted items corresponding to static risk, dynamic need, static protective, and dynamic protective items; using predetermined cut-off scores, individuals are then classified as low-, moderate-, or high- (or none) within each SPIn domain. Notably, domains of criminal history and response to supervision consist of only static items, whereas all other domains (excluding mental health) are based entirely on dynamic factors. Overall, pre-screen risk classifications are also calculated similarly to inform initial risk classification decisions. Only the dynamic need domain ratings and the pre-screen risk classification rating are used in the study.

In the study, one static item from the Criminal History domain (i.e., violent offense conviction) and eight behavioral static items from the Aggression/Violence domain in the SPIn full assessment were used to determine latent class membership (Table 1). Two of the 10 behavioral static items from the Aggression/Violence domain were omitted as both were rated “not applicable” for over 50% of the sample. Dynamic need ratings from the remaining eight SPIn need domains (i.e., substance use, social influences, family, employment, attitudes, social/cognitive skills, stability, and static/dynamic mental health flags) were analyzed to assess for potential differences as a function of class membership (Table 4). Overall pre-screen risk classification ratings from the respondent’s associated SPIn Pre-Screen assessment were also analyzed as a function of class membership.

#### Adverse Childhood Experiences (ACEs)

Felitti et al. (1998) originally identified 10 negative childhood experiences (e.g., witnessing violence in the home) that may increase the likelihood of developing problems such as depression and substance misuse in adulthood; specifically, the greater the number of negative childhood experiences exposed to, the greater the likelihood of developing such problems. The study used a proxy ACE score of seven items taken from the SPIn full assessment: using substances to cope with trauma, coming from a single-parent home, experiencing physical/sexual abuse in family of origin, experiencing violence in the home, experiencing violence/high conflict in the home, instability in the home (i.e., kicked out, foster, or other placements), parental substance abuse problems, and parental mental disorders. Participant total scores ranged from 0 to 7 depending on the presence or absence of each item.

#### Recidivism

Official criminal recidivism information (i.e., any new offense, general or violent) was obtained from Alberta’s Correctional Services division using a fixed 3-year follow-up period. Overall, 27.9% (*n* = 1,054) of the women reported any new charge(s) and 10.6% (*n* = 399) any new violent charge(s) during the fixed 3-year period that commenced after the initial SPIn full assessment.

### Procedure

All requisite ethics clearances were obtained. Next, as described in the participant section, the data was filtered using the following inclusion criteria: (a) women only and (b) the presence of at least one assault/violence offense (previous or current conviction/adjudication) from the SPIn criminal history domain and/or at least one static behavioral indicator from the SPIn aggression/violence domain (from eight possible items) (see Table 1). A resultant sample of 3,773 incarcerated women were retained for analysis.

Maximum likelihood estimation was used to determine class membership using LCA. LCA is a person-centered approach that identifies unmeasured, mutually exclusive subgroups of a population that share common characteristics (Oberski, 2016). For each participant, class membership was determined by score patterns on an observed set of categorical indicators. The SPIn indicators used for inclusion criteria were then used to determine class membership.

Class membership probabilities were then estimated by LCA, reflecting the likelihood that a participant was categorized into the best-fitting class. To determine the number of classes which best fit the data, potential classes are iteratively added to the LCA model. Model fit was then assessed by the following indices: (a) Akaike Information Criterion (AIC) and (b) Bayesian Information Criterion (BIC), where lower fit indices suggest better model fit; (c) the Bootstrap Likelihood Ratio Test (BLRT), where significant *p* values (<.05) are indicative that the current model better fits the data relative to the model with one less class; (d) entropy, where values approaching 1.00 demonstrate greater classification accuracy; (e) Lo–Mendell–Rubin Likelihood Ratio Test (LRT), where significant *p* values (<.05) are indicative that the current model better fits the data, relative to the model with one less class; and (e) classification probabilities, where values approaching 1.00 are indicative of the probability that participants best fit in their currently assigned class. Resultant classes were hypothesized to represent unobservable yet meaningful subgroups of incarcerated women as a function of their violent offending patterns. Finally, a series of chi-square analyses were conducted to assess whether classes differed as a function of their need classifications (i.e., low, moderate, and high) across the eight SPIn need domains (including mental health domain flags), proxy ACE score, pre-screen overall risk classification, and recidivism outcomes.

## Results

LCA was used to determine class membership using Mplus (v8; Muthén & Muthén, 2017). LCA models missing data using maximum likelihood procedures; the command “TYPE = MIXTURE” was used for all analyses. Latent classes were determined by entering the nine indicator variables from the SPIn domains of criminal history and aggression/violence (see Table 1 for study descriptives); the data were then fit to models. Missing data ranged from 0.0% to 29.2% on four of the nine indicators (i.e., violations of no contact or protection orders, violent behavior before age 16, weapon offenses, and history of violence associated with mental disorder). To determine the appropriate number of classes, models ranging from one to five classes were examined; criteria used to assess model fit are discussed in the procedure section.

### Overall Results

Overall results support a three-class model (Table 3). Classification probabilities indicated that the three-class model best fits the data despite slightly larger AIC and BIC values than the four- and five-class models. Though entropy of three- and four-class models were virtually identical, the classification probabilities of the four-class model indicated one class with poor probability that participants were properly classified. Significant *p* values of the Lo–Mendell–Rubin-LRT and BLRT provide additional support for the three-class model.

**Table 3.** Latent Class Analysis: Model Fit Evaluation Information.

Model	AIC	BIC	BLRT	Entropy	LMR–LRT, *p* value	Classification Probabilities
1-Class	37,072.32	37,153.38	—	1.000	—	1 = 1.00
2-Class	36,030.03	36,198.39	−18,523.16*	0.532	.000	1 = 0.85
						2 = 0.85
**3-Class**	**35,407.88**	**35,663.54**	−17,988.02*	**0.752**	**.000**	**1 = 0.93**
						**2 = 0.84**
						**3 = 0.93**
4-Class	35,128.66	35,471.62	−17,662.94*	0.753	.000	1 = 0.83
						2 = 0.89
						3 = 0.94
						4 = 0.64
5-Class	34,997.67	35,427.93	−17,509.33*	0.714	.001	1 = 0.62
						2 = 0.89
						3 = 0.83
						4 = 0.74
						5 = 0.81

*Note.* Bolded values indicate chosen class. AIC = Akaike Information Criteria; BIC = Bayesian Information Criteria; BLRT = Bootstrap Likelihood Ratio Test; LMR–LRT = Lo–Mendell–Rubin Likelihood Ratio Test.

* *p* < .001.

Class membership was assigned to all 3,773 participants. The three resultant classes are best described as: (a) IPV-only women (40.5% of sample; *n* = 1,527); (b) patterned women (19.1% of sample; *n* = 722); and (c) isolated women (40.4% of sample; *n* = 1,524). Chi-square analyses and subsequent *z*-tests of column proportions demonstrated a clear pattern across indicator variables: patterned women predominantly reported the greatest prevalence of criminal history/use of violence indicators, followed by isolated women, then IPV-only women. More specifically, patterned women reported significantly greater prevalence across indicators of assault/violent offense (94.4%), 2 or 3+ previous convictions for violent offenses (36.4% and 50.3%, respectively), violence toward unknown victims (43.8%), violations of no contact or protection orders (12.5%), violent behavior before age 16 (33.9%), at least one weapon offense (38.7%), and any history of violence associated with a mental disorder (11.2%), compared to other groups (Table 4).

**Table 4.** Chi-Square Analyses of Class Differences by LCA Indicator Variables From the SPIn.

Indicator	IPV-Only (40.5%, *n* = 1,527) (%)	Patterned (19.1% *n* = 722)	Isolated (40.4%, *n* = 1,524)	Chi-square	Effect Size (Cramer’s V)	Group Differences^a^
Assault/Violent offense	66.9	94.4	64.9	257.392**	.261	P > IPV = I
Violent behavior in last 6 months	69.9	40.9	32.4	367.952**	.312	IPV > P > I
Previous convictions for violent offense				3,021.385**	.633	
0	40.0	0.0	34.5			IPV > P > I
1	51.7	13.3	61.6			I > IPV > P
2	6.6	36.4	3.9			P > IPV > I
3+	1.7	50.3	0.0			P > IPV > I
Any violence toward unknown victims	0.7	43.8	27.3	698.881**	.430	P > I > IPV
Perpetrator of DV	95.5	43.1	5.1	2,999.062**	.892	IPV > P > I
Violations of protection or no contact orders^b^	8.2	12.5	6.6	25.126**	.082	P > IPV = I
Any violent behavior before 16^c^	3.8	33.9	14.0	279.503**	.311	P > I < IPV
Weapons offenses^d^				366.512**	.259	
0	90.5	61.3	86.8			IPV > I > P
1	9.5	27.0	13.0			P > I > IPV
2	0.0	9.5	0.0			P > IPV = I
3+	0.0	2.2	0.1			P > IPV = I
Any history of violence associated with mental disorder^e^	6.2	11.2	8.9	9.229*	.059	P > IPV^f^

*Note*. DV = domestic violence; IPV = intimate partner violence; LCA = latent class analysis; SPIn = Service Planning Inventory.

a *z*-Test comparisons; I = isolated; IPV = IPV-only; P = patterned.

b 0.0% Missing (*n* = 1).

c 23.6% Missing (*n* = 890).

d 27.7% Missing (*n* = 1,044).

e 29.2% Missing (*n* = 1,100).

f Class I did not significantly differ from either IPV or P.

* *p* < .05. **p < .001.

In contrast, IPV-only women reported significantly greater domestic violence perpetration (95.5%) and violent behavior in the last 6 months (69.9%) compared to other groups. The isolated group had significantly more women with only 1 previous conviction for a violent offense (61.6%) than other groups and did not significantly differ from IPV-only women in terms of the presence of an assault/violent offense, violations of no contact or protection orders, and having 2 or 3+ weapon offenses (Table 4).

### Class Membership as a Function of Risk, Dynamic Need, Recidivism, and ACEs

An additional series of chi-square analyses and subsequent *z*-tests were performed on the dynamic need ratings for each of the eight SPIn need domains (Orbis Partners, 2003), including mental health flags, to determine whether there were significant differences as a function of class membership (Table 5). Patterned women presented the highest level of need in almost all need domains, followed by isolated women and IPV-only women. Specifically, patterned women were significantly more likely to be classified as moderate and high need in regards to substance use (50.4%, 13.6%, respectively), social influences (35.3%, 28.0%, respectively), employment (23.8%, 13.7%, respectively), attitudes (15.0%, 7.6%, respectively), social/cognitive skills (10.0%, 13.0%, respectively), and stability (38.0%, 13.2%, respectively). Notably, the prevalence of high-need family domain women did not statistically differ between IPV-only (37.0%) and patterned women (35.2%), though both groups reported greater prevalence than isolated women (11.7%). Moreover, the prevalence of women with high-need mental health flags was significantly greater among patterned women (56.9%), followed by isolated women (44.6%), then IPV-only women (38.8%).

**Table 5.** Chi-Square Analyses of Class Differences by SPIn Need Domains, Overall SPIn Pre-Screen Risk, and Recidivism Outcomes.

	IPV-Only (40.5%; *n* = 1,527)		Patterned (19.1%; *n* = 722)		Isolated (40.4%; *n* = 1,524)		Chi-square	Effect Size (Cramer’s V)	Group Difference^a^
Category	*n*	%	*n*	%	*n*	%			
**Need domain**									
Substance use							167.91*	.149	
0: None	579	37.9	169	23.4	502	32.9			IPV > I > P
1: Low	358	23.4	91	12.6	254	16.7			IPV > I > P
3: Moderate	535	35.0	364	50.4	658	43.2			P > I > IPV
5: High	55	3.6	98	13.6	110	7.2			P > I > IPV
Social influences							302.02*	.200	
0: None	731	47.9	111	15.4	542	35.6			IPV > I > P
1: Low	301	19.7	154	21.3	296	19.4			IPV = P = I
3: Moderate	380	24.9	255	35.3	443	29.1			P > I > IPV
5: High	115	7.5	202	28.0	243	15.9			P > I > IPV
Family							549.84*	.270	
0: None	76	5.0	81	11.2	404	26.5			I > P > IPV
1: Low	222	14.5	150	20.8	431	28.3			I > P > IPV
3: Moderate	664	43.5	237	32.8	511	33.5			IPV > P = I
5: High	565	37.0	254	35.2	178	11.7			IPV = P > I
Employment							258.27*	.185	
0: None	1,147	75.1	319	44.2	934	61.3			IPV > I > P
1: Low	210	13.8	139	19.3	287	18.8			P = I > IPV
3: Moderate	129	8.4	172	23.8	199	13.1			P > I > IPV
5: High	41	2.7	92	12.7	104	6.8			P > I > IPV
Attitudes							114.88*	.123	
0: None	1,026	67.2	330	45.7	953	62.5			IPV > I > P
1: Low	345	22.6	229	31.7	384	25.2			P > IPV = I
3: Moderate	115	7.5	108	15.0	132	8.7			P > IPV = I
5: High	41	2.7	55	7.6	55	3.6			P > IPV = I
Social/Cognitive skills							179.14*	.154	
0: None	1,211	79.3	403	55.8	1,117	73.3			IPV > I > P
1: Low	211	13.8	153	21.2	230	15.1			P > IPV = I
3: Moderate	68	4.5	72	10.0	99	6.5			P > I > IPV
5: High	37	2.4	94	13.0	78	5.1			P > I > IPV
Stability							241.16*	.179	
0: None	794	52.0	165	22.9	597	39.2			IPV > I > P
1: Low	388	25.4	188	26.0	428	28.1			IPV = P = I
3: Moderate	286	18.7	274	38.0	358	23.5			P > I > IPV
5: High	60	3.9	95	13.2	141	9.3			P > I > IPV
Mental health^b^							67.30*	.094	
1: Low	661	43.3	209	28.9	576	37.8			IPV > I > P
3: Moderate	274	17.9	102	14.1	268	17.6			IPV = I = P
5: High	592	38.8	411	56.9	680	44.6			P > I > IPV
**Pre-screen overall risk score**							771.25*	.320	
1: Low	1,015	66.5	100	13.9	884	58.0			IPV > I > P
3: Moderate	478	31.3	423	58.6	559	36.7			P > I > IPV
5: High	34	2.2	199	27.6	81	5.3			P > I > IPV
**Any recidivism—3 years follow-up**	297	19.4	312	43.2	445	29.2	139.54*	.192	P > I > IPV
**Violent recidivism—3 years follow-up**	128	8.4	128	17.7	143	9.4	49.12*	.114	P > I > IPV

*Note*. IPV = intimate partner violence; SPIn = Service Planning Inventory.

a *z*-Test comparisons. I = isolated; IPV = IPV-only = ; P = patterned.

b Static and dynamic mental health flags.

* *p* < .001.

The theme among classes was also evident in terms of SPIn pre-screen overall risk classification and recidivism outcomes. Patterned women had a significantly greater prevalence of women in the moderate (58.6%) and high (27.6%) pre-screen risk classifications in comparison to isolated women (moderate: 36.7%; high: 5.3%) and IPV-only women (moderate: 31.3%; high: 2.2%). Concerning recidivism, patterned women also reported significantly more general recidivism (43.2%; i.e., any new charge) and violent recidivism (17.7%; i.e., new violent charge) during the fixed 3-year follow-up period, relative to isolated women (general: 29.2%; violent: 9.4%), and IPV-only (general: 19.4%; violent: 9.8%).

In terms of ACEs, Chi-square analyses and subsequent *z*-tests were performed to assess if the presence of proxy ACE items (corresponding to SPIn items) significantly differed as a function of class membership (Table 6). Aligning with previous results, women in the patterned violence group presented the greatest exposure to each of the seven proxy ACE items relative to other groups (*p* < .001), followed by isolated women (*p* < .001) and IPV-only women (*p* < .001).

**Table 6.** Chi-Square Analyses of Class Differences by Proxy ACE items from SPIn Full Assessment.

ACE	IPV-Only (40.5%; *n* = 1,527)		Patterned (19.1%; *n* = 722)		Isolated (40.4%; *n* = 1,524)		Chi-square	Effect Size (Cramer’s V)	Group Difference^a^
	*n*	%	*n*	%	*n*	%			
Uses substances to cope	73	4.8	88	12.2	121	7.9	39.70*	0.103	P > I > IPV
Comes from a single-parent home	316	20.7	328	45.4	504	33.1	150.13*	0.199	P > I > IPV
Experienced physical/sexual abuse	445	29.1	341	47.2	532	34.9	70.56*	0.137	P > I > IPV
Experienced violence in the home	201	13.2	209	28.9	267	17.5	83.26*	0.149	P < I < IPV
Experienced instability in the home	162	10.6	246	34.1	275	18.0	182.04*	0.220	P < I < IPV
Parents used substances	321	21.0	329	45.6	416	27.3	146.83*	0.197	P < I < IPV
Parental mental health issues	52	3.4	59	8.2	84	5.5	23.34*	0.079	P < I < IPV
	*M*	*SD*	*M*	*SD*	*M*	*SD*	*F*	Eta-square	
Total average score	1.03	1.47	2.22	1.74	1.44	1.61	138.42*	0.068	P < I < IPV

*Note*. ACE = adverse childhood experience; IPV = intimate partner violence.

a *z*-Test comparisons. I = isolated; IPV = IPV-only; P = patterned.

* *p* < .001.

## Discussion

Gaining a better understanding of the different risk and need profiles of women categorized as violent is necessary to provide effective treatment and lower the risk of recidivism. Past studies which examined typologies of women who perpetrate violence were largely created using theory-based, researcher-determined decision rules (i.e., Babcock et al., 2003; Kubiak et al., 2013) and/or restricted to women who perpetrate IPV (i.e., Babcock et al., 2003; Fatania, 2010). Thus, our study sought to verify if such theoretically based typologies would also emerge using quantitative clustering (LCA), especially among broader groups of women categorized as violent (i.e., IPV and non-IPV). Further, past research has demonstrated partial support for the RNR model, particularly the risk principle (RNR; Bonta & Andrews, 2024) among justice-impacted women (Blanchette & Brown, 2006; Brown et al., 2020a; Dowden & Andrews, 1999), further examination of how distinct classes of women who engage in violence may differ in terms of recidivism likelihood and, thus, subsequent treatment intensity and supervision requirements was warranted.

Our study used LCA to assess the heterogeneity of women categorized as violent who started community supervision in Western Canada between 2009 and 2012 and were subsequently available during a 3-year fixed follow-up period to assess recidivism. Overall results supported a three-class model: IPV-only women (40.5%, *n* = 1,527), patterned women (19.1%, *n* = 722), and isolated women (40.4%, *n* = 1,524). Many significant differences were observed between groups. Women in the patterned group evidenced the greatest prevalence across most criminal history and aggression/violence indicators, as well as need domains, followed by isolated women, then IPV-only women; this pattern was also evident in overall SPIn pre-screen risk classifications and 3-year follow-up recidivism outcomes.

Overall, women in the patterned violence group greatly differed from women in the isolated and IPV-only groups. Patterned women reported significantly more previous convictions for assault/violent offenses, violence toward unknown victims, violations of protection or no contact orders, violent behavior before age 16, and weapon offenses. This group evidenced greater prevalence of women classified as moderate- and high-risk across criminogenic need domains of substance use, social influences, employment, attitudes, social/cognitive skills, and stability, as well as high-risk mental health flags. Patterned women also had the highest pre-screen overall risk classification and highest rates of general and violent recidivism. Importantly, while patterned women only represented 19% of the sample, it is these women who use violence frequently and across settings who are at heightened risk for continued violence or recidivism, most likely as a consequence of their multiple needs. Thus, women who engage in patterned violence should be afforded the highest levels of treatment intensity (relative to isolated or IPV-only women).

Patterned women, as identified in our study, align with previous typologies of women categorized as violent, including Babcock et al.’s (2003) group of women categorized as generally violent. Both women categorized as patterned and generally violent used violence more frequently and in various contexts (i.e., toward partner and others). Patterned women in our study also align with Kubiak et al.’s (2013) patterned and uncaught typologies; similar to our patterned women, Kubiak et al.’s groups were more likely, relative to other groups, to have been previously arrested or convicted, and to present with greater mental health, substance misuse, and attitude needs. Results of the patterned women group in our study also align with Fatania’s (2010) high-moderate criminality/high-moderate psychopathology typology of women, in that patterned women also evidenced greater criminal history and aggression/violence indicators than other groups, as well as psychopathology risk (i.e., mental health and substance use domains). Taken together, patterned women in our study show similarities with previously identified typologies of women categorized as violent who have been at higher risk (reported the greatest criminal history, use of violence/aggression), and higher need relative to other typologies.

Concerning isolated and IPV-only women, these groups were far more similar to each other than either were to patterned women. Isolated and IPV-only women similarly reported a current assault/violent conviction, and the majority had 0 or 1 previous conviction for a violent offense, though they differed on other variables. IPV women predominantly perpetrated IPV and had no violence toward unknowns, whereas isolated women did not perpetrate IPV (though some reported violence toward unknown victims). IPV-only women also reported more than double the proportion of violent behavior in the past 6 months, compared to isolated women, though this may be due to the target of their violence (domestic) being in close, regular contact with them. In terms of need domains, isolated women reported greater or equal proportions of high-need across all domains (aside from family) in comparison to the IPV-only group. Overall, pre-screen risk and recidivism outcomes (general and violent) were also higher for isolated women than IPV women. This follows logically as isolated women had greater overall needs than IPV-only women.

Similarities can be drawn between IPV-only women and Babcock et al.’s (2003) partner-only women in that violence was only directed toward an intimate partner and not toward unknowns, as well as having lower risk of mental health issues compared to the other group. Similarities can also be drawn to Kubiak et al.’s (2013) isolated group; though most had an assault/violent offense, a large proportion (40%) had no previous convictions for violent offenses. Moreover, like Kubiak et al., our study’s IPV-only group demonstrated the lowest risk for substance misuse, mental health flags, difficulties with social/cognitive skills, and lower overall risk scores. In contrast, isolated women in our study showed no similarities with Babcock et al.’s or Fatania’s (2010) typologies because they did not perpetrate IPV (Babcock and Fatania only used IPV women). The only similarity with Kubiak et al.’s previous isolated group is that the isolated women in our study had the lowest amount of violent behavior in the prior 6 months, and Kubiak et al.’s isolated group also had the least “uncaught” violence in the prior 12 months. More informed comparisons could not be made as the context of the participants’ current offense (s) (i.e., IPV-related or not) was not assessed by Kubiak et al.

Overall, the main difference between findings of our study and past studies concerns victimization history and psychological symptoms. Past typology studies (i.e., Babcock et al., 2003; Kubiak et al., 2013) reported similar or equal rates of past victimization across groups, though groups differed by resultant psychological symptoms. In contrast, groups in our study demonstrated a clear hierarchy in terms of past victimization and resultant psychological symptoms. That is, in the domains that included items of past victimization and psychological symptoms (i.e., mental health flags, family), patterned women had the greatest prevalence of women with high-risk mental health flags and high-risk family needs (e.g., marital risk factors, parenting skills challenges, relational conflict with family) than isolated women; though, patterned women did not significantly differ from IPV-only women in terms of family needs.

Moreover, though patterned women in our study shared similarities with previous typology studies, the IPV-only and isolated groups greatly differed. This could be due to the statistical method used to classify women in our study rather than researcher-derived groupings. Comprehensive assessment of need domains in our study was also able to provide additional participant information that could not be directly compared with past typology studies. Consequently, classes and class differences observed in our study are especially important because they are informed by a broad spectrum of risk/need domains, ACEs, and recidivism outcomes to provide a holistic description of each group of women categorized as violent.

### Trauma Theory and Treatment Implications

A clear theme was evident across the data. Patterned women reported the greatest overall pre-screen risk classification, greatest use of overall aggression/violence, and the highest ratings on dynamic needs (including ACEs), as well as highest general and violent recidivism outcomes. These findings are consistent with trauma theory (Herman, 1992; van der Kolk, 2015). Trauma theory posits that ACEs impact subsequent perceptions and responses to future life events, resulting in negative life outcomes ranging from substance misuse and depression to criminal justice involvement. As an example, childhood trauma has consistently been associated with subsequent offending in adulthood, particularly among groups of women with severely disadvantaged and victimization histories (Cernkovich et al., 2008). Therefore, it is highly likely that the patterned women in our study who used violence frequently and across settings (and who evidenced the highest level of ACEs and SPIn treatment needs) are not only at heightened risk for continued violence or recidivism but would also benefit greatly from trauma-informed interventions.

Two of the most transformational works championing the need for trauma-informed approaches include Harris and Fallot’s (2001), *Using trauma theory to design service systems* and Bloom et al.’s (2003) *Gender-responsive strategies for women offenders: Research, practice, and guiding principles.* Since the publication of these works, various gender-responsive principles have emerged on the global stage from the United Nations Bangkok Rules (2010) to an array of jurisdictional gender-responsive philosophies grounded in the over-arching principle of trauma-informed approach. Similarly, Covington (n.d.) and Van Dieten (n.d.) have been instrumental in developing two decades of trauma-informed programs that meet the needs of justice-impacted women (and girls). In parallel, developments in the neuroscience of trauma, popularized by van der Kolk’s (2015), *The body keeps score* are steadily increasing. Neuroscience-based trauma research has led to the development of third-generation cognitive behavioral therapies that actively integrate traditional elements of cognitive behavioral therapy (CBT) (e.g., changing thinking patterns-alongside the introduction of physical techniques such as deep breathing and yoga) and other therapeutic methods that help people feel safe and grounded—a prerequisite before people can engage in thought-changing work.

Our results also revealed that women in the patterned as well as isolated groups scored higher on ACEs and evidenced behaviors and symptoms consistent with trauma reactions (e.g., mental health issues and alcohol use). Early exposure to abusive and neglectful environments is the context in which many women learn about relationships. For some, reliance on violence and aggressive behavior is normalized within the family and can impact interpersonal expectations and social behaviors throughout the lifespan (Lysova, 2018). Given the high rates of trauma experienced by justice-involved women, it is important to ensure universal precautions. This means that treatment providers and all staff working with women recognize the impacts of trauma, model healthy interpersonal behavior, and respond in a trauma-informed fashion (Substance Abuse and Mental Health Administration Services, 2023).

### RNR-Based Implications

Our results are also consistent with the principles of the RNR framework (Bonta & Andrews, 2024). That is, women who perpetrate violence are neither a homogenous group in terms of needs nor recidivism likelihood. The emphasis of treatment for women identified as low-risk and within the IPV group should include low-intensity intervention characterized by safety planning, increasing awareness of the nature and dynamics of IPV, and introducing alternatives to violence. Women in the low-intensity group may also require access to concurrent interventions; however, an emphasis should be placed on mobilizing natural and mainstream professional supports.

In contrast, women identified as moderate or higher risk with a history of generalized violence (i.e., patterned and isolated) will require more intensive intervention (e.g., more hours) and service options than IPV-only women. In addition to safety planning and increasing IPV awareness and alternatives to violence, women identified as moderate and/or higher risk with generalized violent offending patterns should also receive CBT. The use of CBT to teach effective communication, coping skills, emotional regulation, and interpersonal problem-solving skills have demonstrated favorable outcomes with justice-impacted women (Van Dieten, 2022; Van Dieten et al., 2014). Lastly, women identified as higher risk are also more likely to have multiple or complex needs, including mental health and substance misuse, that should be addressed concurrently or through a continuum of care model.

### Implications for Court-Mandated Treatment Programs

Further implications of these findings are relevant to the use of court-mandated treatment programs for women categorized as violent. Women convicted of IPV-related offenses are often mandated to attend relationship violence intervention programs (RVIP) designed for men (Dutton et al., 2005), regardless of their risk level (e.g., Texas Battering Intervention and Prevention Program, 2013). Thus, women classified as IPV-only or patterned in the current study may both be referred to the same treatment for IPV despite having distinctly different needs underpinning their offending. Of the estimated 2,500 RVIPs across Canada and the United States, over 95% are delivered in male-only groups, which address the needs of men specifically (i.e., male identity) (Canon et al., 2016). Noting the RNR model (Bonta & Andrews, 2024), optimal treatment is achieved when risk level is matched to treatment intensity and treatment targets align with criminogenic needs. However, this is not possible if all women are referred regardless of risk level and/or to male-dominated RVIPs. In turn, some women may be at increased risk for recidivism despite attempted treatment intervention. As victim safety is often dependent on the effectiveness of batterer intervention programs, this crucial implication should be at the forefront of discussion.

### Limitations and Future Directions

Our study was not without limitations. First, the dataset provided did not contain race or ethnicity information beyond Indigenous and non-Indigenous classifications, nor information pertaining to participant socioeconomic status, religion, and sexual orientation. We were, therefore, unable to provide disaggregated findings specific to these aspects of human differences. This limitation specifically concerns issues of generalizability, as sample representativeness in terms of individual/human differences was not able to be assessed. Despite the richness of Canada’s religious and ethnocultural diversity, as well as the large sample size utilized in the current study, the generalizability of results to other countries could not be determined. Second, missing data was observed on four of the nine indicators used to determine class membership: any history of violence associated with mental disorder (29.2%; *n* = 1,100), weapon offenses (27.7%; *n* = 1,044), any violent behavior before age 16 (23.6%; *n* = 890), and violations of protection or no contact orders (0.0%; *n* = 1). Though, despite this, class membership was able to be assigned to all 3,773 participants. Direct comparisons to previous typology studies were also limited as our study utilized full assessments of participant SPIn risk/need domain classification scores (i.e., low-, moderate-, high-risk), whereas past studies opted to select various self-report measures to inform results and may not align with the items assessed in our study. Finally, the use of an archival dataset is a noted limitation. Ideally, if data were collected prospectively, additional interviews or self-report measures could be conducted to further contextualize the quantitative SPIn data. For example, collecting data about the use and frequency of women’s undetected and isolated violence would have provided information needed to make better comparisons with Kubiak et al.’s (2013) uncaught and isolated violence group.

Taken together, future research is needed to assess the additional context that underlies women’s use of violence, both IPV and non-IPV, to better understand the heterogeneity of women categorized as violent. The available research and results of the current study suggest that interventions designed for women should be modified in scope, content, and intensity to reflect the needs of three groups (i.e., IPV, isolated, and patterned). Replications of the current study across other settings (e.g., different security levels and geographical locations) are necessary to further validate the current findings. International replications are of particular interest in terms of treatment implications, as the use of mandated diversion programs in Canada and the United States may not be the same. Finally, the use of mixed-methods (i.e., inclusion of qualitative interviews) is encouraged to deepen our understanding of the context and use of violence, particularly among the group with the greatest risk/need profiles—patterned women.

## Conclusion

Our study adds valuable information to the IPV typology literature. Past studies on typologies of women categorized as violent, determined groupings using researcher-designed decision rules (i.e., Babcock et al., 2003; Kubiak et al., 2013) and/or restricted their samples to women who perpetrate IPV (i.e., Babcock et al., 2003; Fatania, 2010). Therefore, this is the first study to assess the heterogeneity of women categorized as violent, both IPV and non-IPV, using a rigorous quantitative classification method. Although we determined which items to include in the analysis, it was the statistical clustering procedures (rather than us) that determined the number and defining features of the meaningfully distinct classes of women that emerged. Also, to our knowledge, this is the first study to link recidivism outcomes to empirically derived typologies. The large-scale sample used in our study is also able to provide more representative data and results as compared to past typology studies of women categorized as violent.

Results of the LCA and subsequent chi-square analyses of need domains, ACE exposure, and recidivism outcomes demonstrated a clear pattern. The group with the greatest overall dynamic needs, mental health flags, and exposure to ACEs also evidenced the highest recidivism rates. Moreover, the group with the lowest overall dynamic needs, mental health flags, and ACE exposure reported the lowest recidivism outcomes. It can thus be concluded that women with heightened needs in multiple domains—dynamic needs, mental health, trauma history—with an established pattern of using violence inside and outside of intimate partner relationships are at increased risk for recidivism. Such findings are consistent with trauma theory (Herman, 1992) and the RNR model (Bonta & Andrews, 2024). Finally, given that a large proportion (80.9%) of the sample were predominantly low risk, low need, and evidenced low recidivism outcomes, the default starting point for any women with a violent conviction (unless there is evidence of falling in a patterned cluster) should be diversion and the provision of low-intensity community-based intervention, that is trauma-informed and focuses on safety planning, increasing awareness of the nature and dynamics of IPV, and introducing alternatives to violence.

## Notes

1. Seven participants were under age 18 though received an adult sentence.
